# Morphological Covariance and Onset of Foot Prehensility as Indicators of Integrated Evolutionary Dynamics in the Herons (Ardeidae)

**DOI:** 10.1093/iob/obad010

**Published:** 2023-03-22

**Authors:** M F Riegner, R D Bassar

**Affiliations:** Environmental Studies Department, Prescott College, Prescott, AZ 86301, USA; Department of Biological Sciences, Auburn University, Auburn, AL 36849, USA

## Abstract

The ultimate form an organism attains is based, in part, on the rate and timing of developmental trajectories and on compensatory relationships between morphological traits. For example, there is often an inverse correlation between the relative size of an organism's head and the length of its legs. Avian examples with a disproportionately small head and long legs include ostriches (Struthionidae), flamingos (Phoenicopteridae), cranes (Gruidae), and stilts (Recurvirostridae). To determine whether a possible compensatory relationship exists between relative head size and hind-limb length in a typically long-legged family of birds—the Ardeidae—we measured and analyzed skull dimensions (length, width, and height of cranium, and bill length) and skeletal hind-limb dimensions (femur, tibiotarsus, and tarsometatarsus) of the 12 North American species (north of Mexico) and of 12 additional taxa, including the morphologically divergent *Agamia* and *Cochlearius*. We found that *Ardea* species exhibit the smallest relative head sizes associated with the longest legs, while *Butorides, Nycticorax, Nyctanassa*, and *Cochlearius* have among the largest heads relative to hind-limb length. Furthermore, both positive and negative allometries occur in paired comparisons between the three hind-limb bones, expressed in tall morphotypes having disproportionately short femurs while short-legged morphotypes exhibit disproportionately long femurs; we show that this relationship has implications for foraging behavior. Moreover, the nestlings of short-legged herons exhibit functional precociality of the hind limbs through an early onset of prehensile ability of the feet to grasp branches, which is later expressed in adult foraging mode. This developmentally accelerated prehensile function in small-bodied species may be attributed, in part, to selection for predator avoidance in the early nestling stage.

## Abstract (es)

Resumen [Spanish]

La forma final que determina a un organismo esta basada, en parte, por la velocidad y los tiempos de las trayectorias dentro de su desarrollo biológico, asi como en las relaciones compensatorias entre los rasgos morfológicos. Por ejemplo, a menudo existe una correlación inversa entre el tamaño de la cabeza de un organismo en relacion a la longitud de sus patas. Entre algunos ejemplos de aves con cabeza desproporcionadamente pequeña y patas largas se incluyen a las avestruces (Struthionidae), los flamencos (Phoenicopteridae), las grullas (Gruidae) y las cigüeñuelas (Recurvirostridae). Para determinar si existe una posible relación compensatoria entre el tamaño de la cabeza y la longitud de las extremidades traseras en una familia de aves típicas de patas largas—las Ardeidae—medimos y analizamos las dimensiones del cráneo (la longitud, el ancho y la altura), la longitud del pico, asi como las dimensiones esqueléticas de las extremidades posteriores (el fémur, el tibiotarso y el tarsometatarso). Estas medidas fueron aplicadas a las 12 especies de America del Norte (excluyendo a México) y de 12 especies adicionales, incluyendo a morfológicamente divergentes como lo son los *Agamia* y *Cochlearius*. Descubrimos que las especies de *Ardea* que exhiben los tamaños de cabeza más pequeños estan asociados con las patas más largas. Por el contrario, las especies de *Butorides, Nycticorax, Nyctanassa* y *Cochlearius* presentan cabezas más grandes en relación a la longitud de las patas traseras. Asimismo, las alometrías tanto positivas como negativas se presentan en comparaciones pareadas entre los tres huesos de las extremidades posteriores, expresadas en morfotipos altos con fémures desproporcionadamente cortos. Por el contrario, los morfotipos de patas cortas exhiben fémures desproporcionadamente largos, con lo cual mostramos que esta relación contiene implicaciones para el comportamiento en la búsqueda de alimento. Adicionalmente, los polluelos de garzas de patas cortas exhiben una precocidad funcional en las patas traseras a través de un desarollo biologico prematuro en la capacidad prensil de los pies para sujetar las ramas, que posteriormente se expresa en el modo de forrajeo del adulto. Esta capacidad prensil acelerada en especies de cuerpo pequeño, se puede atribuir, en parte, a la selección para evitar a depredadores en esta temprana etapa del desarrollo biologico de la cría.

## Abstract (de)

Kurzfassung [German]

Die endgültige Form, die ein Organismus erreicht, basiert zum Teil auf der Geschwindigkeit und dem zeitlichen Ablauf von Entwicklungsbahnen sowie auf kompensatorischen Beziehungen zwischen morphologischen Merkmalen. Beispielsweise besteht oft eine umgekehrte Korrelation zwischen der relativen Größe des Kopfes eines Organismus und der Länge seiner Beine. Vogelbeispiele mit einem unverhältnismäßig kleinen Kopf und langen Beinen sind Strauße (Struthionidae), Flamingos (Phoenicopteridae), Kraniche (Gruidae) und Stelzen (Recurvirostridae). Um festzustellen, ob bei einer typisch langbeinigen Vogelfamilie—den Ardeidae—eine mögliche kompensatorische Beziehung zwischen der relativen Kopfgröße und der Länge der Hinterbeine besteht, haben wir die Schädelabmessungen (Länge, Breite und Höhe des Schädels und die Schnabellänge) sowie die Skelettmaße der Hinterbeine (Femur, Tibiotarsus und Tarsometatarsus) der 12 nordamerikanischen Arten (nördlich von Mexiko) und von 12 weiteren Taxa, einschließlich der morphologisch abweichenden *Agamia* und *Cochlearius*, gemessen und analysiert. Wir fanden heraus, dass *Ardea*-Arten die kleinsten relativen Kopfgrößen in Verbindung mit den längsten Beinen aufweisen, während *Butorides, Nycticorax, Nyctanassa* und *Cochlearius* die größten Köpfe relativ zur Länge der Hinterbeine haben. Darüber hinaus treten sowohl positive als auch negative Allometrien bei paarweisen Vergleichen zwischen den drei Hintergliedmaßenknochen auf, wobei hochbeinige Morphotypen überproportional kurze Oberschenkelknochen aufweisen, während kurzbeinige Morphotypen überproportional lange Oberschenkelknochen haben. Wir zeigen, dass diese Beziehung Auswirkungen auf das Futtersuchverhalten hat. Darüber hinaus weisen die Nestlinge von kurzbeinigen Reihern eine funktionelle Frühreife der hinteren Gliedmaßen auf, indem die Füße schon früh Äste ergreifen können, was später bei der Futtersuche der Erwachsenen zum Ausdruck kommt. Diese in der Entwicklung beschleunigte Greiffunktion bei Arten mit kleinem Körper kann teilweise auf die Selektion zur Vermeidung von Raubtieren im frühen Nestlingsstadium zurückgeführt werden.

## Introduction

In recent decades, advances in evolutionary developmental biology, or evo-devo (Hall and Olson 2003; West-Eberhard 2003; Carroll 2005), have shed light on the origin and maintenance of morphological diversity in many taxa. A central theme has been that of heterochrony—changes in the rate and timing of development in relation to ancestral developmental trajectories (Gould 1977, 2002; McKinney and McNamara 1991). The evolution of birds from theropod dinosaurs has been attributed to heterochronic outcomes, specifically paedomorphosis (Bhullar et al. 2012, 2016), as has the evolution of toe orientation (Botelho et al. 2015) and even modifications of avian behavior (Andersson 1999).

Traits, however, do not develop in isolation but as integrated units, what Richter and Wirkner (2014) call coherence morphology. Accordingly, compensatory relationships, or trade-offs, exist between and among distinct morphological structures as well as among individual modules (Gatesy and Dial 1996). Thus, “whole morphologies… evolve in a correlated fashion” (Zelditch and Moscarella, 2004, p. 274). This dynamic system of interrelationships was early on articulated by Goethe—the founder of the science of morphology—in his principle of compensation (Lenoir 1987; Riegner 2013). More recently, gene networks have been proposed in which alleles, even at distant loci, exhibit influences across the entire genome, thus more or less affecting all traits (Davidson and Erwin 2006; Yukilevich et al. 2008). In other words, due to widespread pleiotropy, traits lack independence and thus exhibit varying degrees of covariance. Accordingly, West-Eberhard (2003, p. 310) asks: “How often does the hypertrophy of an appendage or organ provoke, or even require, the reduction of some other organ or activity?”

Thus, while traits may covary, they often do not covary in a one-to-one, or isometric, fashion. For example, an inverse correlation between the size of an organism's head and the length of its legs is evident across animal phyla. Among extant birds, a spectrum between small head/long legs and large head/short legs morphotypes is evident. For example, ostriches (Struthionidae) and hummingbirds (Trochilidae) not only occupy opposite regions of avian morphospace with respect to body mass (Pigot et al. 2020), or regarding relative leg vs. wing investment (Heers 2016), but also in cranial and hind-limb proportions. Relative to body size, ostriches have a diminutively small head and exceptionally long legs with two short toes on each foot, while hummingbirds have a disproportionately large head and atrophied legs with four-toed feet. Additional avian examples with a disproportionately small head and long legs include flamingos (Phoenicopteridae), cranes (Gruidae), stilts (Recurvirostridae), Secretarybird (*Sagittarius serpentarius*), Crane Hawk (*Geranospiza caerulescens*), and seriemas (Cariamidae). In contrast, relatively large-headed birds, such as owls (Strigiformes) and parrots (Psittaciformes), typically have short hind limbs and robust feet, which are associated with enhanced foot prehensility (expressed, for example, in the ability to grasp and manipulate prey or other food items; see Backus et al. 2015). Overall, the Passeriformes also have relatively large heads (for their body size), as well as short to mid-length legs and long, prehensile toes, including an elongated hallux especially adapted for grasping perches (Fjeldså et al. 2020). These examples suggest that, while morphological traits—e.g., head size and leg length—typically covary, the nature of this covariance is such that it often deviates from isometry or one-to-one scaling.

Although considered “long-legged wading birds,” there is a wide span (>4.5x) of leg lengths exhibited across the phylogeny of the Ardeidae, from the Zigzag Heron (*Zebrilus undulatus*) to the Goliath Heron (*Ardea goliath*). In addition, even a cursory comparison reveals that cranial dimensions vary from, for example, the slender-headed Tricolored Heron (*Egretta tricolor*) to the relatively robust-headed Yellow-crowned Night-Heron (*Nyctanassa violacea*). Accordingly, the ardeids (hereafter also referred to as “herons”) are ideal subjects to compare and contrast such morphological features to gain insight into the evolutionary dynamics of this taxon, which may hold broad applicability to other taxa.

From an ecomorphological perspective, body size and morphological diversity among herons is associated with variation in foraging behavior and niche occupation (Kushlan 1978). According to Watanabe (2018, p. 2645), the Ardeidae show “prominent interspecific variation in the length of the distal leg even among closely related species, which is associated with foraging behavior…” and their “apparently unique ontogenetic trajectory might have facilitated diversification of distal leg length and hence foraging habitat segregation in this family.” For example, taller birds are able to forage at greater water depths (McKilligan 2005, p. 34; Morales 2018) and capture larger prey than relatively short-legged species. Furthermore, while taller species, for example, the Reddish Egret (*E. rufescens*), regularly hunt by active wading (Koczur et al. 2020) or, as in the Great Egret (*A. alba*), may walk slowly across wide expanses, shorter-legged taxa, such as the Green Heron (*Butorides virescens*), often perch on roots or branches and wait for prey to come within striking range, a relatively low-energy hunting strategy (Meyerriecks 1960; Kushlan 1978; Kushlan and Hancock 2005).

Since hind-limb length is a critical determinant of ardeid foraging ecology, the drivers of differential limb growth, and associated morphological and behavioral adaptations, are worthy of investigation. For example, there is evidence the mechanism for differential growth of ardeid hind-limb bones can be attributed to varying rates of cell proliferation, resulting in “morphological heterochrony” (Cubo et al. 2000). Furthermore, Ávila (2011, 2017), in studies of growth rates in seven species of ardeids, identified heterochrony or, more specifically, hypermorphosis, as “the main evolutionary process in… Ardeidae,” resulting from a relatively extended postnatal growth period (Ávila 2011, p. 771), which culminates in *Ardea*. For example, tarsometatarsus growth in the Green Heron terminates already on Day 20 while in the Great Egret termination is delayed until Day 43 (Ávila 2011). Between these extremes are the Black-crowned Night-Heron (*Nycticorax nycticorax*), at Day 23, and the Reddish Egret, at Day 36 (Ávila 2011). Thus, differential limb growth durations are expressed in paedomorphic, or relatively “underdeveloped,” and peramorphic, or relatively “overdeveloped,” heterochronic patterns evident in short-legged vs. long-legged herons, respectively.

The behavioral elements, and certainly the morphological components, of heron foraging behavior presumably originate in early ontogeny, in some cases perhaps in the very early nestling developmental phase of these mostly semi-altricial birds. Kushlan and Hancock (2005) identified the “brancher” stage as the age at which heron nestlings first clamber from the nest—an early developmental marker indicating when the prehensile ability of the feet is first exhibited. As discussed later, we propose that foot prehensility plays a significant role in the foraging repertoire of various heron species.

In this study, we test whether morphological characters of herons deviate from isometry—perhaps indicative of morphological trade-offs—and whether this deviation is related to the foraging behaviors of adults and to the timing of first appearance of nascent foraging behavior components in nestlings. Specifically, we address the following questions: (1) Do heron species with longer legs have disproportionately smaller heads (negative allometry) while, conversely, species with shorter legs have disproportionately large heads? (2) Do the skeletal components of the hind limbs scale isometrically as overall leg length among species increases or is differential growth evident (positive or negative allometry)? (3) Does the degree of deviation from isometry among the three hind-limb bones correlate with foraging behavior? (4) Do heron species with relatively short legs exhibit an enhanced foot prehensility (as alluded to earlier in regard to short-legged owls and parrots), perhaps already evident during the nestling stage?

Our working definition of morphological trade-off, as implied by the measurement of negative allometries, presupposes that an organism is a dynamic integrated whole, and (assuming constant body mass) that “an increase in the magnitude of one [trait] means a decrease in the magnitude of one or more others” (West-Eberhard 2003, p. 296). In other words, “the simplest type of trade-off occurs when one trait cannot increase without a decrease in another” (Garland et al. 2022, p. 83). Furthermore, “most traits can be considered to involve a trade-off with every other trait, since the development or performance of every trait involves some cost in terms of time, material, or energy and therefore may detract from others” (West-Eberhard 2003, p. 303). Acknowledging that “trade-off” defies clear definition, Garland et al. (2022) identify six nonmutually exclusive categories of trade-offs and argue for a pluralistic perspective that embraces multiple levels of biological organization across disciplinary boundaries. By attempting to integrate morphology, behavior, development, and evolution, our study lends support to Garland et al.’s perspective.

And finally, why test for covariance and potential morphological trade-offs specifically between the limb module and the cranium module? More typically, comparisons are made between/among avian “locomotor modules,” such as the forelimbs, hind limbs, and tail (e.g., Gatesy and Dial 1996; Eliason et al. 2023). However, just as one can apply a logical conceptual model of “locomotor modules” to avian anatomy, so too can one identify “foraging modules,” which, especially in the herons, would embrace both cranial and hind-limb morphologies.

## Materials and methods

### Morphological measurements

We took morphological measurements of 24 species of Ardeidae from museum skeletal specimens (37.5% of 64 extant species; Winkler et al. 2015), including all 12 North American species north of Mexico (Table 1). Additionally, 12 other taxa were selected to represent a broad range of sizes and morphologies and had accessible specimens housed in United States natural history collections (see Acknowledgments for list of institutions). We attempted to measure a minimum of 10 samples per species but were not always able to do so due to the rarity of some specimens. We measured a total of 265 adult specimens, though 15 specimens were incomplete (e.g., a skull may have been intact, but a femur was missing). We measured all skull dimensions (width, height, and length of cranium, bill length, and total skull length (= cranium length + bill length; Table S1) and skeletal hind-limb lengths (femur, tibiotarsus, and tarsometatarsus; Table S2) using a Mitutoyo 4LB11 digital caliper (± 0.01 mm). We used a ruler (± 1.0 mm) to measure some hind-limb bones of the largest specimens (e.g., *A. goliath*) that were too long for the caliper (>160 mm). Cranial length was measured from the nasofrontal hinge along the occipital crest to the greatest distance of the occipital bone, and cranial width was measured as the distance between the distal ends of the two post-orbital processes. Bill length was measured from the nasofrontal hinge to the distal tip of the bill. Hind-limb dimensions were measured as follows: femur, from the tip of the trochanter to the distal medial condyle; tibiotarsus, from the cnemial crest to the distal medial condyle; and tarsometatarsus, from the calcaneal ridge to the distal trochlea for digit III. One person (M.F. Riegner) took all the measurements.

**Table 1:** Species included in this study and sample size. Taxonomy, taxonomic order, and English spelling, here and throughout, follow Chesser et al. (2022) (with the exception of the Whistling Heron) and Clements et al. (2022)

Species common name	Scientific name	Sample size
American Bittern	*Botaurus lentiginosus*	13
Zigzag Heron	*Zebrilus undulatus*	3
Least Bittern	*Ixobrychus exilis*	14
Bare-throated Tiger-Heron	*Tigrisoma mexicanum*	12 (1 incomplete)
Great Blue Heron	*Ardea herodias*	13
Cocoi Heron	*A. cocoi*	9 (2 incomplete)
Goliath Heron	*A. goliath*	8
Great Egret	*A. alba*	12
Pacific Reef-Heron	*Egretta sacra*	14 (1 incomplete)
Snowy Egret	*E. thula*	14
Little Blue Heron	*E. caerulea*	12
Tricolored Heron	*E. tricolor*	14 (1 incomplete)
Reddish Egret	*E. rufescens*	15 (4 incomplete)
Black Heron	*E. ardesiaca*	5
Cattle Egret	*Bubulcus ibis*	12
Squacco Heron	*Ardeola ralloides*	10 (1 incomplete)
Green Heron	*Butorides virescens*	12
Agami Heron	*Agamia agami*	3
Whistling Heron	*Syrigma sibilatrix*	15 (3 incomplete)
Capped Heron	*Pilherodius pileatus*	12 (2 incomplete)
Black-crowned Night-Heron	*Nycticorax nycticorax*	14
Yellow-crowned Night-Heron	*Nyctanassa violacea*	13
Malayan Night-Heron	*Gorsachius melanolophus*	2
Boat-billed Heron	*Cochlearius cochlearius*	14
	Total =	265

Body mass data for each of the 24 species were obtained from the literature (Dunning 2008; Table S3); when multiple values were given, such as for males and females, means were calculated (following Zeffer et al. 2003).

### Behavioral indices

Foraging behavior diversity and activity-level categories were determined from publications, especially Kushlan (1978), but also Martínez-Vilalta and Motis (1992) and Kushlan and Hancock (2005), and Cornell Lab of Ornithology Birds of the World online, as well as from M.F. Riegner’s field observations, over several decades, of 18 of the 24 species included herein. Four categories were estimated and scored, ranging from category 1, representing minimal behavioral diversity and generally stationary perching/crouching, to category 4, representing relatively high behavioral diversity and high activity, including running and turning quickly (column E, Table 2). We then compared foraging activity level with allometric scaling of the femur, which was proportionately the most variable hind-limb bone.

**Table 2:** Ardeid foraging behavior diversity

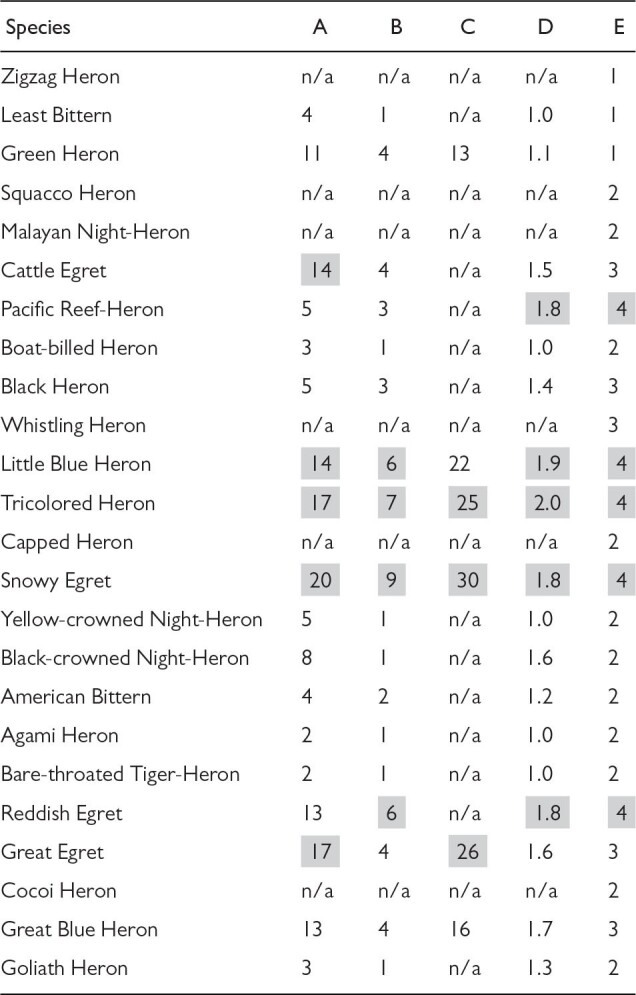

Column A: foraging behavior diversity tabulated from Kushlan (1978, Table 1)

Column B: foraging behavior diversity adjusted to include only ambulatory behaviors

Column C: additional foraging behavior diversity data from Kelly et al. (2003)

Column D: foraging (i.e., feeding) activity indexes from Kushlan (1978, Fig. 2)

Column E: estimated foraging activity-level categories: (1) perching and “passive;” (2) standing, crouching, “inactive,” and “motionless;” (3) slow wading, slow walking, standing, and patrolling large area; (4) “active,” running, and turning quickly. Estimates tabulated from data and accounts from Kushlan (1978), Martínez-Vilalta & Motis (1992), and Kushlan & Hancock (2005), Cornell Lab of Ornithology Birds of the World online, and personal observations; terms in quotes are actual descriptors taken from the preceding sources from individual species accounts. Species ordered by increasing total leg length. Shaded boxes indicate the three most diverse foraging behaviors or foraging activity indexes within a column (except Column E where only the highest category is shaded), which tend to be displayed in mid-sized species; n/a = not available.

Regarding the age when nestlings first clamber from the nest (the “brancher” stage), data were taken from the literature, as well as provided by various heron field researchers. There is a range from approximately 5–21 Days, and possibly beyond, for the onset of this developmental marker (Table 3). We compared the age of the brancher stage with allometric scaling of leg length and, when descriptions were available, we identified which species as adults tend to forage by grasping branches, reeds, mangrove roots, etc. We considered these species as expressing a relatively high degree of foot prehensility in their adult foraging repertoire, especially compared with those species that typically forage by walking on firm substrate, wading in shallow water, standing on mud flats, etc. Of course, all ardeids are capable of perching on branches to roost, etc.

**Table 3:** Approximate age at which nestlings first stand and clamber from the nest (the “brancher” stage)

Common name	Age in days	References
American Bittern	7–14^1^ (n/a)	Lowther et al. (2020); Baicich & Harrison (1997)
Zigzag Heron	n/a^1^ (n/a)	Mathews & Brooke (1988)
Least Bittern	4–5^1^ (16%)	Nero (1950); Weller (1961); Baicich & Harrison (1997); Corman (2005)
Great Blue Heron	21 (40%)	Pratt (1970)
Gray Heron^2^	25–27 (54%)	Voisin (1991)
Goliath Heron	21 (50%)	Kushlan & Hancock (2005)
Purple Heron^2^	20 (45%)	Tomlinson (1975)
Great Egret	21 (42%)	Baicich & Harrison (1997); Kushlan & Hancock (2005); Wise-Gervais (2005c)
Intermediate Egret^2^	20–23 (50%)	McKilligan (2005); M. Mashiko (personal communication)
Snowy Egret	10–21 (28%)	McVaugh (1972); Baicich & Harrison (1997); Burger (2005)
Little Blue Heron	12–13 (34%)	McVaugh (1972); Werschkul (1979); Baicich & Harrison (1997)
Tricolored Heron	11–17 (44%)	McVaugh (1972); Baicich & Harrison (1997); Frederick (2020)
Reddish Egret	14 (44%)	M.C. Green (personal communication)
Cattle Egret	14/15 (47%)	Kushlan & Hancock (2005)/Voisin (1991); Wise-Gervais (2005b)
Squacco Heron	14 (40%)	Kushlan & Hancock (2005)
Green Heron	7^1^ (32%)	Baicich & Harrison (1997); Kushlan & Hancock (2005)
Agami Heron	n/a^1^ (n/a)	Kushlan & Hines (2016); A. Stier (personal communication)
Black-crowned Night-Heron	10–14 (25%)	McVaugh (1972); Chapman et al. (1981); Voisin (1991); Baicich & Harrison (1997); Wise-Gervais (2005a)
Yellow-crowned Night-Heron	21 (57%)	Bagley and Grau (1979)
Boat-billed Heron	7–8^1^ (28%)	Kushlan (2009); P. Baldovinos Rogel (personal communication)

1 Species in which adults display high degree of foot prehensility by typically grasping branches, reeds, mangrove roots, snags, etc., while foraging or, in the case of the American Bittern, grasping vertical reeds when resting between foraging bouts. Numbers in parentheses are rough estimates of % of total nestling period (up to fledging) when the brancher stage first occurs. Fledging estimates are from species accounts in Cornell Lab of Ornithology Birds of the World online; n/a = not available.

2 Species that are not part of this study's dataset but are included here because information on brancher stage was accessible in the literature.

### Statistical analyses

We first tested whether the relationship between body mass and other traits (leg length, cranium length, cranium width, cranium height, bill length, and total skull length) differed from what is expected based on isometric scaling of a volumetric measurement (using mass as a proxy for volume) and length measurements of the other traits. Such an approach is appropriate because we are interested in the covariation between body size, morphological traits, and foraging behaviors and not how morphological traits and foraging behaviors are related independently of the effect of body size. We log_10_ transformed both body mass and the other variables and included log_10_ body mass as a predictor in a general linear model (GLM) with the other traits, one at a time, as dependent variables. Based on allometric scaling expectations, volumetric traits (V) and length traits (L) are predicted to scale to the one-third, based on $V\ = \ a{L}^3$ or $L = {(\frac{1}{a}V)}^{1/3}$. On the log_10_ scale, this equation is ${\log}_{10}\, L = - {\rm{lo}}{{\rm{g}}}_{10}a\ + \frac{1}{3}{\rm{lo}}{{\rm{g}}}_{10}V$, meaning that isometry between overall size and length measurements are expected to result in slope parameters that are not different than one-third. We tested for this relationship using ${\rm{lo}}{{\rm{g}}}_{10}$-transformed linear measurements of the leg lengths and cranium dimensions as dependent variables and ${\rm{lo}}{{\rm{g}}}_{10}$-transformed body mass as a predictor variable in a GLM. We asked whether the estimated relationship between length and mass on the log_10_ scale differed from one-third using a *t*-test. Slope parameters that were significantly lower than one-third were considered to show negative allometric relationships and those that were significantly greater than one-third were considered to show positive allometric relationships. We centered the analysis on the mean log_10_ mass across the species.

Next, to test the hypothesis that heron species with longer legs have disproportionately smaller heads (negative allometry), we conducted a similar analysis to the one above using overall leg length as an independent variable and cranium length, cranium width, cranium height, bill length, and total skull length as dependent variables. Each of these traits is a length measurement and thus an isometric relationship would be indicated by slope parameters of 1. We asked whether the estimated relationship between these traits on the log_10_ scale differed from 1 using a GLM and tested whether the estimated slope parameter differed from 1 using a *t*-test.

We then asked whether there was evidence for allometric relationships between the constituent bones of the leg. We did so by including total leg length as a predictor in the GLM and the length of the individual leg bones (femur, tibiotarsus, and tarsometatarsus) as dependent variables. As in the relationships between leg length and cranium dimensions, isometric scaling is expected to produce a slope parameter of 1 between the individual bones of the leg.

To ask whether the degree of deviation from isometry of the femur to the overall leg length relates to foraging behavior, we subtracted the expected femur length based on an isometric relationship from the observed femur length of each species and used this as a measure of deviation from isometry. Species with positive values of this measure have longer femurs than expected based on their leg length and species with negative values have shorter femurs for their leg length than based on isometric scaling. We then used these values as dependent variables in a GLM model with our four behavior categories as categorical predictors. We asked if there was evidence for species with different foraging behaviors to have different deviations of femur length from isometry using an omnibus *F*-test. If the *F*-test was significant, we then asked which of the behavior categories were different from each other using a three-contrast post-hoc test (1 vs. 2, 2 vs. 3, and 3 vs. 4).

We used a similar approach to ask whether herons with relatively shorter legs exhibit functional precociality through an accelerated foot prehensile ability, that is, an early expression of the brancher stage. Here, we used the deviation from the allometric relationship between total leg length and mass as a dependent variable and used age (days post-hatching) at brancher stage as a continuous predictor.

We performed both nonphylogenetically informed and phylogenetically informed analyses using program R (R Core Team 2020). Nonphylogenetic analyses were conducted using the *lm* function (R Core Team 2020). For the phylogenetically informed analyses, we based our phylogeny on Hruska (2018). However, a few species in *Ardea* were not included in Hruska's phylogeny so we supplemented the information for this genus from Huang et al. (2016; Fig. S1). We then performed the analyses using the *gls* function (Pinheiro et al. 2020) and assumed Brownian motion with correlation structures calculated using *corBrownian* (Paradis and Schliep 2019).

To increase sample size, as well as to include incompletely labeled specimens, data for adult males and females were combined (following Zeffer et al. 2003; Maccarone and Brzorad 2016).

## Results

### Do larger (i.e., heavier-bodied) herons have smaller heads and longer legs than expected based on allometric scaling?

**Fig. 1:**
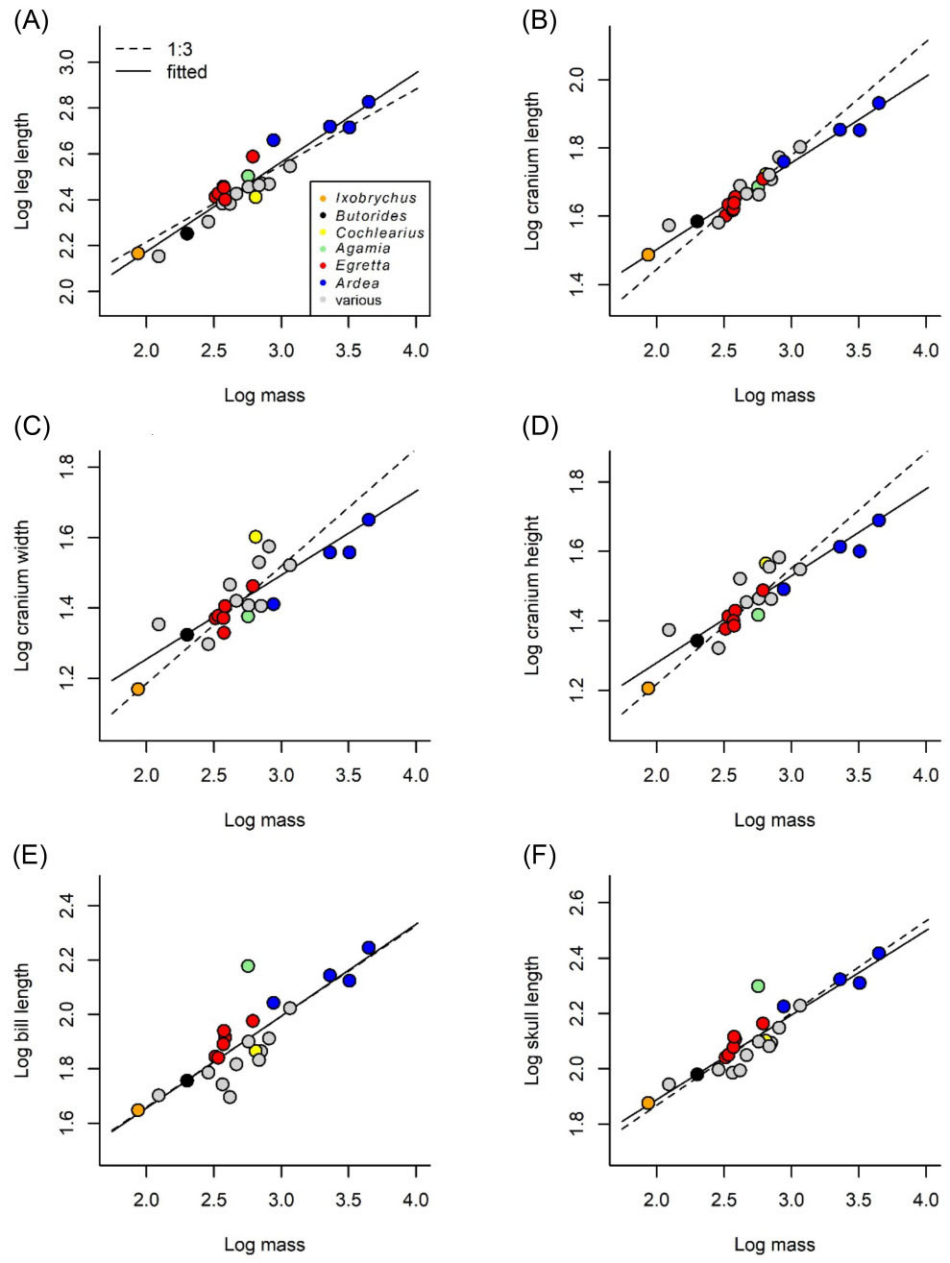
Regression plots of nonphylogenetically corrected lengths of skull characters against body mass. (**A**) leg length; (**B**) cranium length; (**C**) cranium width; (**D**) cranium height; (**E**) bill length; and (**F**) total skull length. Fit lines are those from the nonphylogenetic regressions. Dashed lines give the expected relationship based on isometric (1:3) scaling.

Larger (heavier) heron species have larger heads and longer legs (all *P* < 0.001, Table 4); significant correlation between avian body mass and limb measurements is expected (Eliason et al. 2023). However, cranium dimensions and leg length scaled in different ways to body mass. Larger herons have longer legs than expected based on isometric scaling (positive allometry), although this deviation from isometry was not significant with the number of species analyzed (nonphylo: ${t}_{22} = 1.98,\ P\ = \ 0.060$ and phylo: ${t}_{22} = 1.43,\ P\ = \ 0.166$; Fig. 1A). In contrast, three measures of cranium dimensions were smaller than expected based on isometric scaling such that larger herons have smaller craniums than expected (negative allometry) (cranium length: ${t}_{22} = 6.30,\ P < 0.001;\ $cranium width: ${t}_{22} = 2.99,\ P\ = \ 0.007$; and cranium height: ${t}_{22} = 3.36,\ P\ = \ 0.003$; Fig. 1B–D). This deviation was the case even after controlling for phylogeny (cranium length: ${t}_{22} = 5.21,\ P < 0.001;\ $cranium width: ${t}_{22} = 3.06,\ P\ = \ 0.006$; and cranium height: ${t}_{22} = 3.19,\ P\ = \ 0.004$). The only two exceptions were that bill length (i.e., culmen) and total skull length (= cranium length + bill length) scaled isometrically with body size (bill length: nonphylo: ${t}_{22} = 0.09,\ P\ = \ 0.927$ and phylo: ${t}_{22} = 0.75,P\ = \ 0.459$; Fig. 1E and total skull length: nonphylo: ${t}_{22} = 0.930,\ P\ = \ 0.362$ and phylo: ${t}_{22} = 1.91,P\ = \ 0.069$; Fig. 1F). Notable divergent morphologies are apparent in *Cochlearius* for cranium width (yellow point, Fig. 1C) and *Agamia* for bill length and total skull length (green points, Fig. 1E and F, respectively).

**Table 4:** Parameter estimates, standard errors, and significance from nonphylogenetic and phylogenetic regressions of log_10_-transformed character lengths against log_10_-transformed body mass. Phylogenetic regression assumed Brownian motion. Degrees of freedom for all tests was 22

	Nonphylogenetic analyses				Phylogenetic analyses			
Parameter	Estimate	SE	*t*	*P*	Estimate	SE	*t*	*P*
**Leg length**								
Intercept	2.46	0.011	226.4	<0.001	2.44	0.048	50.63	<0.001
Mass	0.39	0.028	13.81	<0.001	0.37	0.026	14.44	<0.001
**Cranium width**								
Intercept	1.43	0.012	116.2	<0.001	1.46	0.058	25.19	<0.001
Mass	0.24	0.032	7.47	<0.001	0.24	0.031	7.78	<0.001
**Cranium height**								
Intercept	1.46	0.010	152.5	<0.001	1.48	0.047	31.27	<0.001
Mass	0.25	0.025	10.03	<0.001	0.25	0.025	10.04	<0.001
**Cranium length**								
Intercept	1.69	0.005	345.3	<0.001	1.70	0.031	54.21	<0.001
Mass	0.25	0.013	19.98	<0.001	0.25	0.017	14.75	<0.001
**Bill length**								
Intercept	1.90	0.018	107.8	<0.001	1.91	0.069	27.8	<0.001
Mass	0.34	0.046	7.4	<0.001	0.31	0.037	8.4	<0.001
**Skull length**								
Intercept	2.11	0.011	186.0	<0.001	2.13	0.049	43.04	<0.001
Mass	0.31	0.029	10.39	<0.001	0.28	0.026	10.77	<0.001

### Do heron species with longer legs have disproportionately smaller heads (negative allometry) while, conversely, species with shorter legs have disproportionately larger heads?

**Fig. 2:**
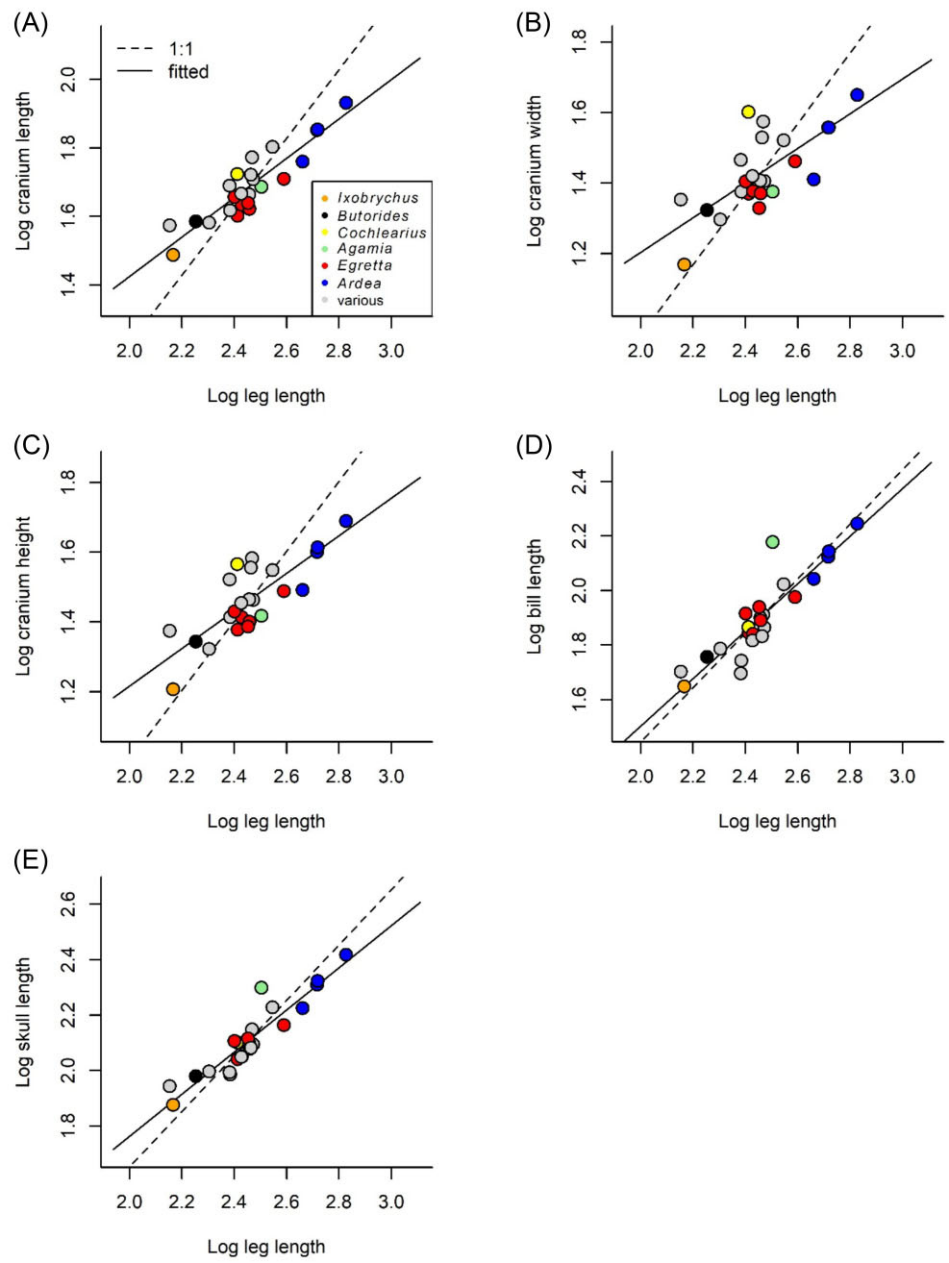
Regression plots of nonphylogenetically corrected lengths of skull characters against leg length. (**A**) cranium length; (**B**) cranium width; (**C**) cranium height; (**D**) bill length; and (**E**) total skull length. Fit lines are those from the nonphylogenetic regressions. Dashed lines give the expected relationship based on isometric scaling (1:1).

When we directly compared the allometry between the length of the legs and the dimensions of the head, these differences in the allometric scaling became clearer (Table 5, Fig. 2). The relationship of cranium dimensions to leg length showed considerable negative allometry, with longer-legged birds having smaller than expected craniums (cranium length: ${t}_{22} = 7.51,P < 0.001;\ {\rm{Fig}}.{\rm{\ }}2{\rm{A}}$; cranium width: ${t}_{22} = 5.01,P < 0.001;\ {\rm{Fig}}.{\rm{\ }}2{\rm{B}}$; cranium height: ${t}_{22} = 5.42,\ P < 0.001;\ {\rm{Fig}}.{\rm{\ }}2{\rm{C}};$ and total skull length: ${t}_{22} = 3.76,\ P\ = \ 0.001;$Fig. 2E). This was the case even after controlling for phylogeny (cranium length: ${t}_{22} = 6.88,P < 0.001$; cranium width: ${t}_{22} = 4.61,\ P < 0.001$; cranium height: ${t}_{22} = 4.84,P < 0.001$; and total skull length: ${t}_{22} = 4.00,P\ = \ 0.001$). Regarding bill length, a component of total skull length, there was no difference from isometry based on nonphylogenetic analysis (nonphylo: ${t}_{22} = 1.39,P\ = \ 0.178;$Fig. 2D), while significant negative allometry was apparent when phylogeny was controlled (phylo: ${t}_{22} = 2.27,P\ = \ 0.034$), thus indicating that taller species have shorter than expected bill lengths. Notable divergent morphologies are again evident in *Cochlearius* for cranium width (yellow point, Fig. 2B) and *Agamia* for bill length and total skull length (green points, Fig. 2D and E, respectively).

**Table 5:** Results from nonphylogenetic and phylogenetic regressions of log_10_-transformed head dimensions against log_10_-transformed leg length. Phylogenetic regression assumed Brownian motion. Degrees of freedom for all tests was 22

	Nonphylogenetic analyses				Phylogenetic analyses			
Parameter	Estimate	SE	*t*	*P*	Estimate	SE	*t*	*P*
**Cranium width**								
Intercept	1.43	0.016	88.78	<0.001	1.47	0.068	21.47	<0.001
Leg length	0.49	0.102	4.84	<0.001	0.57	0.093	6.09	<0.001
**Cranium height**								
Intercept	1.46	0.013	108.5	<0.001	1.49	0.059	25.38	<0.001
Leg length	0.54	0.085	6.33	<0.001	0.61	0.08	7.59	<0.001
**Cranium length**								
Intercept	1.69	0.009	188.2	<0.001	1.71	0.042	41.18	<0.001
Leg length	0.58	0.057	10.16	<0.001	0.61	0.057	10.69	<0.001
**Bill length**								
Intercept	1.90	0.015	129.5	<0.001	1.93	0.066	29.3	<0.001
Leg length	0.87	0.093	9.39	<0.001	0.80	0.090	8.85	<0.001
**Skull length**								
Intercept	2.11	0.010	208.5	<0.001	2.14	0.050	42.50	<0.001
Leg length	0.76	0.064	11.89	<0.001	0.72	0.069	10.52	<0.001

### Do the three skeletal components of the hind limbs scale isometrically as overall leg length increases or is differential growth evident (positive or negative allometry)?

**Fig. 3:**
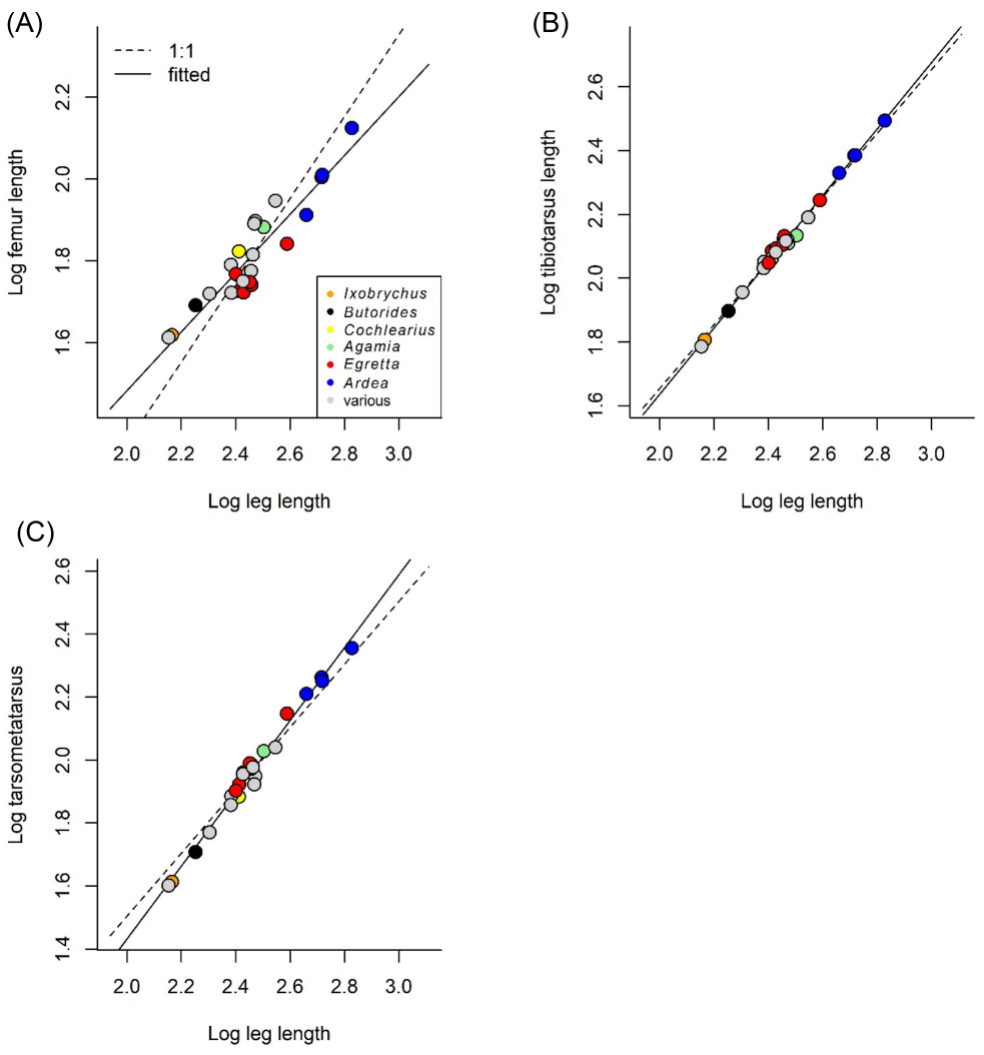
Regression plots of nonphylogenetically corrected leg bone lengths against total leg length. (**A**) femur; (**B**) tibiotarsus; and (**C**) tarsometatarsus. Fit lines are those from the nonphylogenetic regressions. Dashed lines give the expected relationship based on isometric (1:1) scaling.

Herons with longer legs had shorter femurs than expected based on isometric scaling (negative allometry) (nonphylo: ${t}_{22} = 4.54,P < 0.001$ and phylo: ${t}_{22} = 3.22,P\ = \ 0.004$; Table 6, Fig. 3A). However, both the tibiotarsus and the tarsometatarsus compensated for some of this negative allometry in the overall length of the leg, as both showed positive allometry with overall leg length (tibiotarsus: ${t}_{22} = 2.81,\ P\ = \ 0.010$ and tarsometatarsus: ${t}_{22} = 5.08,P < 0.001$; Fig. 3B and C, respectively). Including the phylogenetic relationships in the analysis altered the results for the tibiotarsus (${t}_{22} = 0.680,P\ = \ 0.504$) but not for the tarsometatarsus (${t}_{22} = 3.49,\ P\ = \ 0.002$).

**Table 6:** Results from nonphylogenetic and phylogenetic regressions of log_10_-transformed leg bone lengths against log_10_-transformed total leg length. Phylogenetic regression assumed Brownian motion. Degrees of freedom for all tests was 22

	Nonphylogenetic analyses				Phylogenetic analyses			
Parameter	Estimate	SE	*t*	*P*	Estimate	SE	*t*	*P*
**Femur**								
Intercept	1.81	0.010	184.4	<0.001	1.85	0.039	47.71	<0.001
Total leg length	0.72	0.062	11.58	<0.001	0.83	0.053	15.69	<0.001
**Tibiotarsus**								
Intercept	2.12	0.002	920.7	<0.001	2.11	0.011	189.3	<0.001
Total leg length	1.04	0.014	71.81	<0.001	1.01	0.015	66.4	<0.001
**Tarsometatarsus**								
Intercept	1.96	0.005	402.8	<0.001	1.95	0.024	83.15	<0.001
Total leg length	1.16	0.031	37.58	<0.001	1.11	0.032	34.62	<0.001

### Is the degree of deviation from isometry of the femur associated with foraging behavior?

**Fig. 4:**
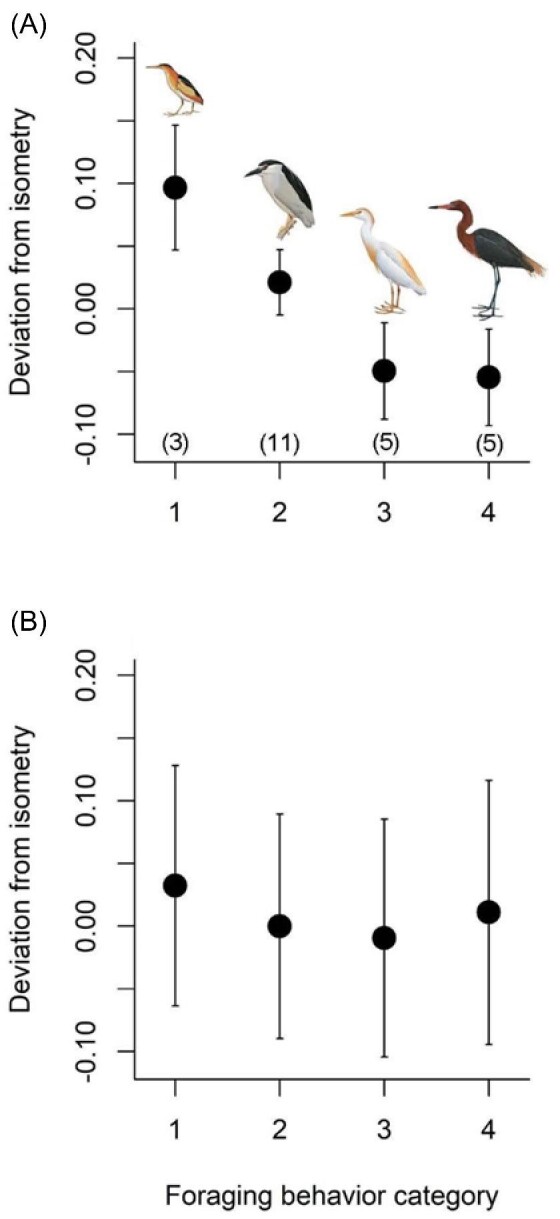
Relationship between the deviation of femur length from isometry with leg length and behavioral mode without (**A**) and with (**B**) phylogenetic corrections. Points are means and bars are 95% confidence intervals. See Table 2, column E, for estimation of foraging activity level and species assigned to each category; numbers in parentheses indicate sample size in each foraging activity-level category. Representative species from each foraging activity-level category (from left to right): Least Bittern (*Ixobrychus exilis*), Black-crowned Night-Heron (*Nycticorax nycticorax*), Cattle Egret (*Bubulcus ibis*), and Reddish Egret (*Egretta rufescens*). Bird illustrations reproduced with permission from Lynx Edicions.

The deviation in the length of the femur from isometry was associated with foraging behavior (${F}_{3,20} = 10.38,\ P < 0.001$; Fig. 4A). Species that displayed foraging behavior activity-level category 1 (i.e., the lowest activity level, such as perch-and-wait) had significantly more positive deviations from isometry than species in category 2 (${F}_{1,20} = 6.96,P\ = \ 0.016$; Fig. 4A). In contrast, category 3 species had significantly more negative deviations from isometry than category 2 species (${F}_{1,20} = 8.89,P\ = \ 0.007$; Fig. 4A). Species with foraging behavior activity-level category 4 did not differ from species assigned to category 3 (${F}_{1,20} = 0.03,\ P\ = \ 0.857$; Fig. 4A). These associations between foraging behavior activity level and deviations of the femur from isometry were due almost entirely to phylogeny (${F}_{3,20} = 1.13,\ P\ = \ 0.361$; Fig. 4B).

### Do nestlings of small heron species with relatively short legs exhibit an early onset of foot prehensility, that is, an early expression of the brancher stage?

**Fig. 5:**
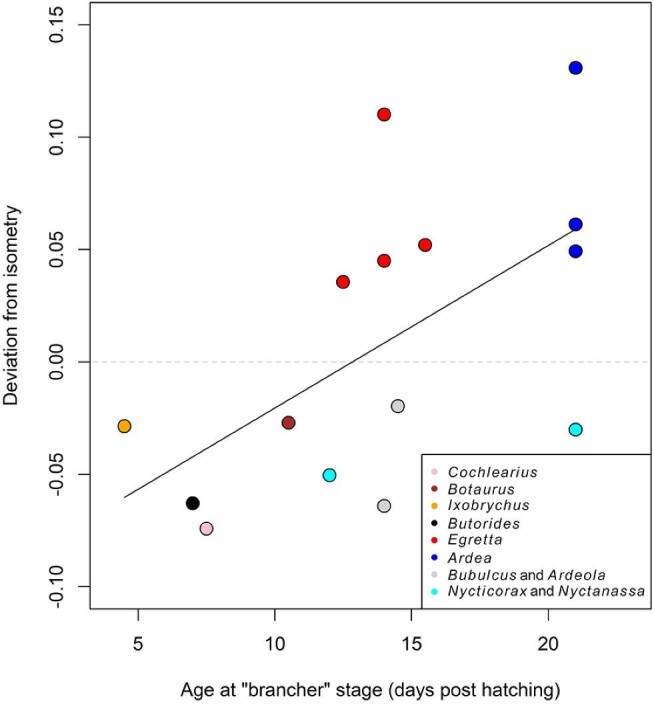
Relationship between the deviation of total leg length from isometry with body mass against age at brancher stage, with fitted line (data for brancher stage from Table 3; if range was reported, then mean was calculated). Those points below the horizontal dashed line indicate species that have shorter than expected total leg lengths for a given body mass in contrast to points above the dashed line that indicate species with longer than expected total leg lengths for a given body mass. The latter tend to take relatively more days to attain the brancher stage.

Species with shorter than expected legs relative to body mass, compared with isometric scaling, tended to be younger (e.g., Day 7 posthatching in the Green Heron) when first exhibiting grasping ability (nonphylo: ${t}_{12} = 2.66,\ P\ = \ 0.022$ and phylo: ${t}_{12} = 1.84,\ P\ = \ 0.089$; Table 3, Fig. 5); these results are attributed to phylogeny. Those species that exhibit a delayed development of foot prehensility, for example, Day 21, are among the tallest of herons (e.g., *Ardea* herons, dark blue points, Fig. 5). The Yellow-crowned Night-Heron (rightmost light blue point) has shorter legs than expected for a delayed brancher stage. Finally, the onset of foot prehensility is relatively delayed until up to 40–54% of the total nestling period has transpired in the large *Ardea* herons, while this developmental marker is accelerated to only 16% of the total nestling period in the Least Bittern and 32% in the Green Heron (Table 3). In other words, not only does the brancher stage first appear in relatively fewer days in the smaller ardeids, but also it appears proportionately much sooner in their respective overall nestling periods compared with the longer-legged herons.

## Discussion

### Cranium and hind-limb morphological relationships

**Fig. 6:**
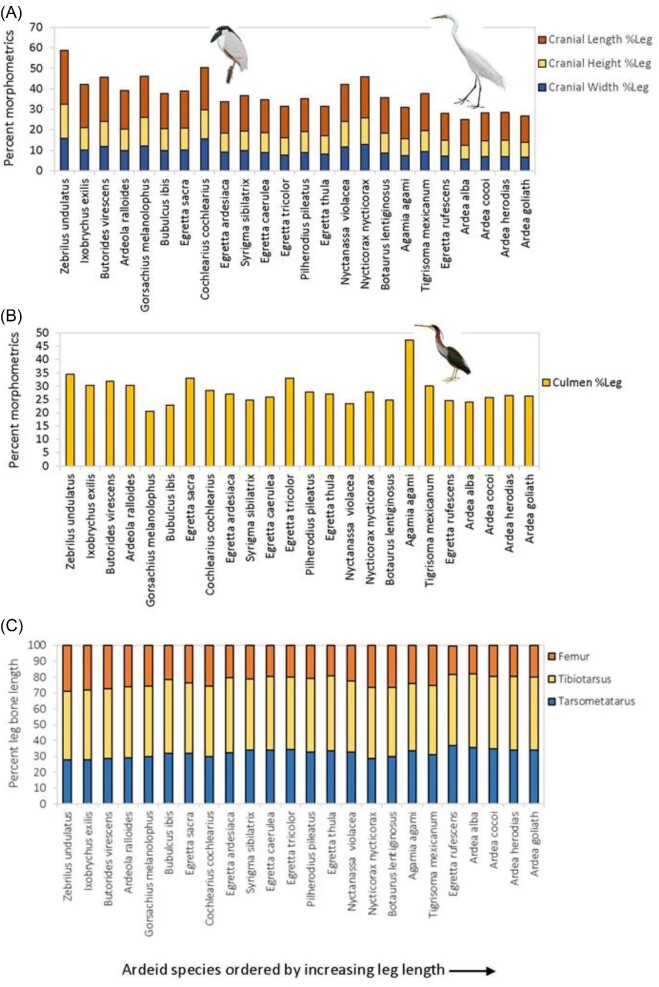
Ardeid morphometrics. (**A**) Cranium dimensions as % total leg length (note: percentages are not additive); (**B**) culmen length as % total leg length; (**C**) Leg bone lengths as % total leg length. Species represented are Boat-billed Heron (*Cochlearius cochlearius*), Great Egret (*Ardea alba*), and Agami Heron (*Agamia agami*). Bird illustrations reproduced with permission from Lynx Edicions.

We found significant departures from isometry between cranial dimensions and length of hind limbs in ardeids. The tallest species generally exhibit the smallest relative head dimensions, with *A. alba* as an extreme morphotype in this regard (Fig. 6A). In contrast, *Cochlearius* exhibits among the most robust head dimensions relative to leg length (Fig. 6A) and in this respect represents the “opposite” ardeid morphotype to *A. alba*. In other words, *Cochlearius* and *A. alba* can be considered as occupying divergent extremes of ardeid morphospace. Clearly, however, the overall morphological trajectory can be somewhat diverted, presumably due to strong selection pressure resulting in specialized adaptation and niche occupation, as evident in the exceptionally long, slender bill and total skull length of *Agamia* (green points in Fig. 1E and F and Fig. 2D and E; Fig. 6B) or the heavy, robust cranium to accommodate the broad, scoop-like bill of *Cochlearius* (yellow points in Figs 1C and 2B; Fig. 6A).

### Intra-hind-limb morphological relationships

Regarding possible trade-offs between hind-limb elements, the two distal hind-limb segments—tibiotarsus and tarsometatarsus—are responsible for the greatest contribution to increase in total leg length in herons, whereas the femur becomes disproportionately shorter in longer-legged species (Table S2, Fig. 6C). Similarly, in a study of 323 diverse species of birds, increase in leg length was attributed mainly to the distal bones, i.e., tibiotarsus and tarsometatarsus, and not to the femur (Zeffer et al. 2003, p. 467): “relatively long-legged birds have relatively shorter femurs compared with short-legged species.” Compared with the norm for all birds (that is, the 323 species measured in their study), waders have a significantly lower mean value for femur index but significantly higher values for tibiotarsus and tarsometatarsus. Barbosa and Moreno (1999) maintain that relative lengthening of the tibiotarsus and tarsometatarsus results in increased stride length in “waders” (i.e., shorebirds), while increase in relative femur length translates into increased stride frequency. Moreover, Zeffer et al. (2003) measured proportionately longer femurs in birds of prey and hanging and climbing species, both of which have relatively well-developed prehensile feet; in our study, disproportionately longer femurs were associated with small, short-legged heron species (e.g., Least Bittern), which exhibit a functional precociality of the grasping ability carried over into the adult morphofunctionality (see next section).

As in our study, Stoessel et al. (2013) found a positive correlation between tibiotarsus and tarsometatarsus lengths among 236 species of birds sampled across all major avian subclades, but an “inverse proportionality” between the femur and tarsometatarsus (as also stated for flamingos by Eliason et al. 2023, p. 348); notably, in addition, an inverse proportional covariation between the femur and tibiotarsus was determined only in “rather long-legged birds” (p. 489), as our data indicate for 24 heron taxa. Overall, the avian hind-limb bones clearly exhibit a high degree of integration for efficient terrestrial locomotion (Stoessel et al. 2013).

### Onset of foot prehensility (the “brancher” stage)

As shown in this study, foot prehensility develops earlier in species with relatively shorter hind limbs (and thus longer femurs), that is, in smaller morphotypes, which typically employ perching and crouching in the adult foraging repertoire. What may be a driver for the evolution of functional precociality of the hind limbs in smaller species of herons, subsequently displayed in typical foraging modes in adults? In general, small species with accelerated foot prehensility, e.g., Green Heron, are mostly solitary or loosely colonial nesters (Davis and Kushlan 2020), and/or nest close to the ground or just above water (e.g., Least Bittern nests in emergent vegetation only 15–76 cm above the water's surface: Poole et al. 2020; Zigzag Heron, of which there is little known, is also a solitary nester, as is the American Bittern: HeronConservation 2020). Thus, nestlings of these species are more vulnerable to predators (e.g., lack “safety in numbers”) compared with densely colonial species and/or those that nest higher in trees; they also typically have smaller body sizes and consequently would be susceptible to a broader array of predators. Therefore, it behooves nestlings of these species to accelerate their ability to clamber from the nest at an early stage of development in order to escape predators. Researchers who band nestling herons can attest that, as soon as young birds are capable of scrambling from the nest, they will climb away when approached. Selection may therefore act on the early nestling stage to accelerate a behavioral ability that is then retained and expressed in the foraging behavior of the adult.

Carrier (1996, p. 480), investigating the ontogeny of locomotor behavior in vertebrates, maintains that, due to “higher predation pressure on juveniles, the locomotor phenotype of adults may, in some cases, be more a reflection of selection acting on the performance of juveniles than a direct result of selection acting on the adults” (see also Herrel and Gibb 2006; Young et al. 2022), what Gignac and Santana (2016) call “ontogenetic inertia.” For example, in Mallards (*Anas platyrhynchos*), “hindlimbs retain characteristically juvenile morphology and performance” (Dial and Carrier 2012, p. 3707). Thus, the proclivity of certain species of herons—e.g., Least Bittern and Green Heron—to forage while grasping a perch, may be attributed to mostly solitary nesting near the ground (or water surface), concomitant greater vulnerability to predation, and the evolution of functional precociality of foot prehensility to enable early-stage nestlings to clamber from the nest to safety. In other words, “vulnerability to predation would lead to selection for accelerated development” (Carrier 1996, p. 480). While the early onset of foot prehensility in early-stage nestlings is an expression of ontogenetic acceleration, this juvenile feature subsequently remains imprinted on the foraging behavior repertoire and is expressed throughout the adult life stage. And finally, although the brancher stage has not been previously studied systematically in herons, or in most avian taxa, Holcomb (1966) examined the development of the grasping reflex in seven species of passerines, a taxon noted for the evolution of enhanced prehensility resulting in specialized perching ability (Fjeldså et al. 2020). The American Goldfinch (*Carduelis* [*Spinus*] *tristis*) showed the earliest expression of prehensility at Day 5 post hatching while the other species ranged to Day 9 (Holcomb 1966). Accordingly, among ardeids, the Least Bittern converges with passerines in this early developmental marker (Table 3).

### Morphological trade-offs?

The evolution of the avian *Bauplan* has been marked by repeated morphological trade-offs and compensations. For example, the forelimbs evolved aerodynamic ability at the expense of the hind limbs, as evidenced in relative limb lengths and associated muscle mass (Heers and Dial 2012, 2015; Heers 2016). Furthermore, as proavian forelimbs lengthened for flight and hind limbs shortened, the former relinquished grasping ability, while the latter evolved prehensility (Heers and Dial 2012). Among herons, our results show that shorter-hind-limbed species exhibit an accelerated development of the grasping reflex and a frequent application of prehensility in adult foraging behavior. This trend, in fact, may echo the evolution of perching in the transition from nonavian maniraptoran dinosaurs to birds. Dececchi and Larsson (2013) note that the hind limbs of *Archaeopteryx* were relatively shorter (by at least 30%) than those of similar-sized *Microraptor* (the approximate proportional difference between, respectively, Green Heron [*B. virescens*] and Little Blue Heron [*E. caerulea*]), and that “long limbs would hinder perching and movement along tree branches” (p. 2750). Apparently, the adaptive shortening of avian hind limbs permits a concomitant enhancement of prehensility.

In other examples of compensatory evolutionary responses in birds, Harell (2016) identified a negative correlation *within* the hind-limb module regarding the lengths of the cnemial crest vs. the patella, the former shorter and the latter longer in cormorants in comparison with anhingas. Bhullar et al. (2016) proposed a morphological and functional compensation driven by heterochrony between the loss of hand functions of dinosaurs, as hands were integrated into the avian wing, and the enhanced kinematic functions of the avian beak; however, kinematic grasping function of feet was not considered in their formula. Regarding morphological integration and compensations specifically between and among avian hind-limb elements, Stoessel et al. (2013, p. 483) summarize it nicely: “Different demands on posture, locomotor mechanics, or behavior are known to influence differences in length between intra-limb elements, particularly overall proportions.”

As mentioned earlier, we consider the avian hind limbs and cranial features as “foraging modules,” and thus the negative allometric relationships between these two modules, identified in this study, can be considered expressions of the integrated nature of the whole organism, an expanded evolutionary perspective garnering renewed attention these days (e.g., Schwenk et al. 2009; Huneman 2010; Nicholson 2014). Even in early studies, reciprocal relationships were demonstrated between what were initially thought to be independent structures, such as comparisons of tarsus length and pointedness of bill in icterids (James 1982). More recently, correlated evolution was demonstrated between, for example, avian sternal keel length and ilium length (Zhao et al. 2017), thus indicating that the avian organism expresses a high degree of integration (see Orkney et al. 2021), with morphological compensations occurring at various levels of organization. Relationships between apparently disparate organs, perhaps through pleiotropic effects, seem to be the rule rather than the exception across organisms, as evidenced, for example, in a single set of genes controlling both limb and horn development in *Onthophagus* beetles (Moczek 2005). Our study was limited in its focus to only skeletal features; however, especially regarding foot prehensility, avian muscle distribution would also need examination (Backus et al. 2015), as well as accommodations of the nervous system, blood supply, etc.

Although neck length was not measured in our study, Böhmer et al. (2019, p. 5) found that avian “neck length showed an isometric scaling in relation to total leg length,” and thus the same can be assumed for ardeids: that is, longer-legged herons should have longer necks, and thus longer-necked herons should have disproportionately smaller heads. This relationship is evident in a cursory comparison of, for example, relatively large-headed, short-necked *Nycticorax* and *Nyctanassa* night-herons with relatively small-headed, long-necked *Ardea* and *Egretta* day-herons. Accordingly, longer-necked herons would be expected to have disproportionately shorter femurs; in fact, Böhmer et al. (2019) identify the Gray Heron (*A. cinerea*) as having an exceptionally long neck in relation to femur length.

### Brancher stage, hind-limb length, relative femur length, and foraging behavior

**Fig. 7:**
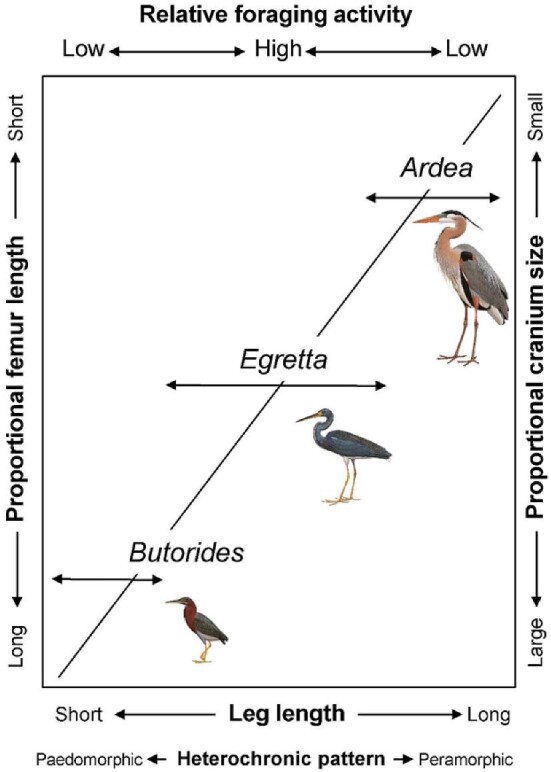
Diagrammatic representation of ardeid hypothetical morphospace inferred from interrelationship of total leg length, leg heterochronic growth pattern, proportional femur length, proportional cranium size, and relative foraging activity. Representative species are (from left to right) Green Heron (*Butorides virescens*), Tricolored Heron (*Egretta tricolor*), and Great Blue Heron (*Ardea herodias*). Bird illustrations reproduced with permission from Lynx Edicions.

Species that display the earliest development of the brancher stage, e.g., the small-bodied Least Bittern and Green Heron (Table 3), and, we propose, most likely the Zigzag Heron, possess short legs with longer than expected femurs (Table S2, Figs 3A, 5, and 6C) and are those in which the adults tend to employ a minimal diversity of low-energy foraging behaviors (category 1, Table 2, Figs 4, and S1C and D), such as crouch-/perch-and-wait, by grasping branches, reeds, mangrove roots, snags, etc. (Meyerriecks 1960; Kushlan and Hancock 2005). Species that exhibit the most delayed development of foot prehensility, from Day 21 and beyond, i.e., the tall *Ardea* herons (Table 3), exhibit long legs with shorter than expected femurs (Table S2, Figs 3A, 5, and 6C) and are those species in which the adults, compared with the smallest heron species, display relatively more foraging behaviors, including low-energy behaviors, such as stand-and-wait/peering over, as well as slightly more active behaviors, including slow wading while patrolling broad swaths of habitat in search of prey (categories 2 and 3, Table 2, Figs 4, and S1C, D; Kushlan and Hancock 2005). Notably, unlike, for example, the Green Heron, such tall morphotypes, specifically *A. alba* and *A. cocoi*, “rarely use perches to forage” (Pinto et al. 2013, p. 793). Species that develop prehensility at intermediate ages (Day 10–20 posthatching), e.g., the *Egretta* herons (Table 3), possess mid-length legs (∼260–300 mm) with shorter than expected femurs (Table 3, Figs 3A, 5, and 6C) and are those in which adults display the most diverse and most active foraging behaviors (mostly category 4, Table 2, Figs 4, and S1C, D), such as running, turning quickly, foot-stirring, wing-flicking, etc. (Kushlan 1978; Kushlan and Hancock 2005). Morales (2018) also found that *Egretta* species display more active foraging behaviors than *Ardea* species (regarding feeding behavior diversity and body size, see also Meyerriecks 1960, p. 149, and Kushlan 1978, p. 255). Accordingly, in our sample, the intermediate-sized herons exhibit the highest species richness as well as the highest diversity of foraging behaviors, including the most active foraging behaviors (Table 2, Fig. S1C, D). Thus, by considering relative timing of hind-limb functional development, intra-hind-limb morphological relationships, proportional relationships of cranium size, and elements of foraging behavior within an ecomorphological context, an approximation of ardeid hypothetical morphospace can be inferred that defines the possibilities and boundaries of potential form within this avian clade (Fig. 7).

## Conclusions

In conclusion, our study interrelated (1) potential morphological trade-offs between cranial and hind-limb modules, (2) variation in the relative proportions of intra-hind-limb elements, (3) association of foraging behavior with hind-limb morphology, (4) variation in the early development of the hind-limb grasping reflex, and (5) possible selection for predator avoidance in early ontogeny in smaller-bodied taxa. Accordingly, these ecomorphological relationships may contribute to integrated evolutionary dynamics that ultimately influence ardeid morphospace, morphofunctionality, and adaptation to a broad range of habitats and foraging circumstances in this widely distributed group of wading birds.

## Supplementary Material

obad010_Supplemental_Files

## Data Availability

The data underlying this article are available at https://doi.org/10.5061/dryad.wwpzgmspq.
