# Supplementary material for: Morphological Covariance and Onset of Foot Prehensility as Indicators of Integrated Evolutionary Dynamics in the Herons (Ardeidae)
Source: Integr Org Biol. 2023 Mar 22;5(1):obad010. doi: 10.1093/iob/obad010 (PMC10132848; doi:10.1093/iob/obad010)
Supplement: obad010_Supplemental_Files [file obad010_supplemental_files.zip › Heron Morphology.Table S1.IOB-2022-051.R1.docx]

**Table S1** Summary statistics for ardeid cranium dimensions^1^.

| Species | Cranium width | Cranium height | Cranium length | Total skull length |
| --- | --- | --- | --- | --- |
| American Bittern | 24.2-26.6 | 24.6-31.7 | 47.9-55.7 | 115.7-133.4 |
| (*n =* 13) | 25.5 ± 0.7 | 29.1 ± 1.9 | 51.1 ± 2.5 | 124.4 ± 6.2 |
|  | 8.6% | 9.8% | 17.2% | 42.0% |
| Zigzag Heron | 16.6% | 22.9-24.5 | n/a | n/a |
| (*n =* 3) | 22.6 ± 1.0 | 23.7 ± 1.1 | 37.5 | 87.9 |
|  | 15.9% | 16.6% | 26.3% | 61.7% |
| Least Bittern | 14.2-15.4 | 14.2-17.5 | 29.4-32.1 | 69.8-82.7 |
| (*n =* 14) | 14.8 ± 0.4 | 16.1 ± 0.9 | 30.7 ± 0.8 | 75.2 ± 3.1 |
|  | 10.1% | 11.0% | 20.9% | 51.3% |
| Bare-throated Tiger-Heron | 32.0-35.3 | 34.0-36.8 | 59.8-66.8 | 145.1-192.0 |
| (*n =* 12) | 33.2 ± 0.5 | 35.4 ± 0.8 | 63.6 ± 1.9 | 169.1 ± 13.0 |
|  | 9.4% | 10.1% | 18.1% | 48.1% |
| Great Blue Heron | 33.3-37.5 | 38.1-44.9 | 65.9-74.6 | 192-235 |
| (*n =* 13) | 36.1 ± 1.3 | 41.1 ± 1.8 | 71.5 ± 2.7 | 210.8 ± 13.4 |
|  | 6.9% | 7.9% | 13.7% | 40.3% |
| Cocoi Heron | 34.5-37.9 | 38.4-41.9 | 68.2-75.8 | 195-224 |
| (*n =* 9) | 36.2 ± 1.3 | 39.9 ± 1.0 | 71.2 ± 2.3 | 204.2 ± 8.5 |
|  | 7.0% | 7.7% | 13.7% | 39.2% |
| Goliath Heron | 40.5-47.7 | 46.8-51.6 | 80.9-90.2 | 243-277 |
| (*n =* 8) | 44.7 ± 2.2 | 48.9 ± 1.4 | 85.5 ± 3.2 | 261.9 ± 12.2 |
|  | 6.7% | 7.3% | 12.7% | 39.0% |
| Great Egret | 23.9-30.3 | 27.4-33.5 | 55.4-59.7 | 159-178 |
| (*n =* 12) | 25.7 ± 1.6 | 31.1 ± 2.1 | 57.6 ± 1.3 | 168.0 ± 6.1 |
|  | 5.6% | 6.8% | 12.6% | 36.7% |
| Pacific Reef-Heron | 22.7-26.8 | 24.7-28.5 | 42.1-48.7 | 113.3-136.2 |
| (*n =* 14) | 25.4 ± 1.2 | 26.9 ± 1.1 | 45.4 ± 1.8 | 128.0 ± 7.1 |
|  | 10.1% | 10.7% | 18.1% | 50.9% |
| Snowy Egret | 21.8-25.0 | 22.8-26.5 | 39.1-44.6 | 103.4-129.4 |
| (*n =* 14) | 23.5 ± 0.8 | 25.2 ± 1.3 | 41.9 ± 1.6 | 119.8 ± 7.6 |
|  | 8.2% | 8.8% | 14.6% | 41.7% |
| Little Blue Heron | 22.3-24.7 | 23.6-27.5 | 41.3-45.6 | 101.6-122.5 |
| (*n =* 12) | 23.9 ± 0.7 | 26.0 ± 1.3 | 43.1 ± 1.1 | 112.5 ± 5.8 |
|  | 8.9% | 9.7% | 16.1% | 41.9% |
| Tricolored Heron | 19.9-22.6 | 22.3-26.1 | 41.4-46.4 | 100.9-143.3 |
| (*n =* 14) | 21.4 ± 0.7 | 24.4 ± 1.0 | 43.6 ± 1.3 | 130.7 ± 10.8 |
|  | 7.6% | 8.6% | 15.4% | 46.2% |
| Reddish Egret | 26.7-30.9 | 28.4-33.2 | 44.0-54.7 | 132.1-158.9 |
| (*n =* 15) | 29.0 ± 1.2 | 30.8 ± 1.4 | 51.2 ± 2.8 | 145.9 ± 6.2 |
|  | 7.5% | 7.9% | 13.2% | 37.6% |
| Black Heron | 22.9-23.9 | 23.0-24.8 | 38.3-41.3 | 100.4-124.3 |
| (*n =* 5) | 23.5 ± 0.4 | 23.9 ± 0.8 | 40.0 ± 1.1 | 110.1 ± 9.9 |
|  | 9.1% | 9.2% | 15.5% | 42.6% |
| Cattle Egret | 23.0-24.7 | 22.6-27.8 | 40.0-42.9 | 92.5-103.6 |
| (*n =* 12) | 23.8 ± 0.6 | 25.9 ± 1.5 | 41.5 ± 0.9 | 96.8 ± 3.3 |
|  | 9.8% | 10.7% | 17.1% | 40.0% |
| Squacco Heron | 18.0-21.1 | 18.5-23.3 | 34.9-40.4 | 86.4-107.2 |
| (*n =* 10) | 19.9 ± 1.0 | 21.0 ± 1.6 | 38.2 ± 1.8 | 99.4 ± 5.9 |
|  | 9.9% | 10.4% | 18.9% | 49.3% |
| Green Heron | 19.7-23.0 | 21.1-23.1 | 36.8-40.8 | 82.8-104.6 |
| (*n =* 12) | 21.1 ± 1.0 | 22.1 ± 0.6 | 38.5 ± 1.2 | 95.6 ± 6.2 |
|  | 11.8% | 12.3% | 21.5% | 53.4% |
| Agami Heron | 22.8-24.4 | 25.7-27.1 | 47.4-49.5 | 198.0-202.0 |
| (*n =* 3) | 23.8 ± 0.9 | 26.2 ± 0.8 | 48.6 ± 1.1 | 199.3 ± 2.3 |
|  | 7.5% | 8.2% | 15.2% | 62.5% |
| Whistling Heron | 25.1-27.7 | 25.4-30.3 | 43.3-49.3 | 105.5-120.4 |
| (*n =* 15) | 26.3 ± 0.8 | 28.5 ± 1.4 | 46.4 ± 1.4 | 112.1 ± 4.5 |
|  | 9.9% | 10.7% | 17.4% | 42.0% |
| Capped Heron | 24.5-26.8 | 27.6-30.7 | 44.6-48.3 | 118.3-134.7 |
| (*n =* 12) | 25.6 ± 0.7 | 29.2 ± 1.0 | 46.2 ± 1.2 | 125.6 ± 5.3 |
|  | 8.9% | 10.2% | 16.1% | 43.9% |
| Black-crowned Night-Heron | 32.8-40.0 | 35.4-40.6 | 55.6-62.7 | 129.3-148.9 |
| (*n =* 14) | 37.6 ± 2.2 | 38.3 ± 1.8 | 59.3 ± 2.2 | 140.9 ± 5.8 |
|  | 12.8% | 13.0% | 20.2% | 47.9% |
| Yellow-crowned Night-Heron | 32.1-36.1 | 31.7-39.6 | 49.5-57.4 | 112.0-134.4 |
| (*n =* 13) | 33.9 ± 1.3 | 36.0 ± 2.3 | 52.7 ± 2.1 | 120.8 ± 6.5 |
|  | 11.6% | 12.4% | 18.1% | 41.5% |
| Malayan Night-Heron | 28.7-29.8 | 32.5-34.0 | 48.4-49.5 | 97.0-100.4 |
| (*n =* 2) | 29.3 ± 0.8 | 33.3 ± 1.1 | 49.0 ± 0.8 | 98.7 ± 2.4 |
|  | 12.1% | 13.8% | 20.3% | 40.9% |
| Boat-billed Heron | 37.0-43.5 | 33.5-40.5 | 49.1-56.8 | 113.3-138.4 |
| (*n =* 14) | 40.0 ± 2.0 | 36.8 ± 1.7 | 52.9 ± 2.4 | 126.3 ± 7.4 |
|  | 15.5% | 14.3% | 20.5% | 49.0% |

^1^Values are range (in mm), mean with standard deviation (in mm), and mean percent (mean ratio x 100) of total leg length for each cranium dimension per each species. Right column: range (in mm) and mean with standard deviation (in mm) of total skull length (cranium length + bill length) per species.
