# Supplementary material for: Morphological Covariance and Onset of Foot Prehensility as Indicators of Integrated Evolutionary Dynamics in the Herons (Ardeidae)
Source: Integr Org Biol. 2023 Mar 22;5(1):obad010. doi: 10.1093/iob/obad010 (PMC10132848; doi:10.1093/iob/obad010)
Supplement: obad010_Supplemental_Files [file obad010_supplemental_files.zip › Heron Morphology.Table S2.IOB-2022-051.R1.docx]

**Table S2** Summary statistics for ardeid hind-limb bone lengths^1^.

| Species | Femur | Tibiotarsus | Tarsometatarsus | Total leg length |
| --- | --- | --- | --- | --- |
| American Bittern | 74.2-83.5 | 121.5-137.5 | 84.2-94.8 | 282.3-315.8 |
| (*n =* 13) | 78.8 ± 3.2 | 128.6 ± 5.1 | 89.0 ± 3.1 | 296.4 ± 10.9 |
|  | 26.6% | 43.4% | 30.0% |  |
| Zigzag Heron | 39.8-41.9 | 60.9-61.3 | 36.5-43.2 | 138.9-146.0 |
| (*n =* 3) | 40.9 ± 1.1 | 61.1 ± 0.3 | 40.0 ± 3.4 | 142.5 ± 5.0 |
|  | 28.7% | 42.9% | 28.1% |  |
| Least Bittern | 40.0-43.3 | 61.2-66.7 | 38.4-48.9 | 140.3-155.8 |
| (*n =* 14) | 41.5 ± 1.0 | 64.0 ± 1.5 | 41.1 ± 2.5 | 146.6 ± 4.1 |
|  | 28.3% | 43.7% | 28.0% |  |
| Bare-throated Tiger-Heron | 78.5-94.0 | 127.3-169.0 | 91.6-117.5 | 297.4-379.5 |
| (*n =* 12) | 88.4 ± 4.5 | 154.9 ± 10.3 | 109.6 ± 6.8 | 351.6 ± 21.3 |
|  | 25.5% | 44.1% | 31.2% |  |
| Great Blue Heron | 94.8-110.3 | 221-268 | 155-202 | 471.8-574.6 |
| (*n =* 13) | 102.2 ± 5.3 | 243.2 ± 17.5 | 178.0 ± 15.1 | 523.4 ± 36.8 |
|  | 19.5% | 46.5% | 34.0% |  |
| Cocoi Heron | 98.1-104.3 | 213-267 | 158-203 | 475.8-574.3 |
| (*n =* 9) | 101.8 ± 2.0 | 242.6 ± 19.3 | 183.1 ± 17.0 | 520.3 ± 37.1 |
|  | 19.4% | 46.6% | 35.2% |  |
| Goliath Heron | 124.8-139.3 | 265-348 | 192-247 | 581.8-733.3 |
| (*n =* 8) | 133.1 ± 5.0 | 311.8 ± 30.3 | 227.0 ± 18.4 | 671.8 ± 51.4 |
|  | 19.8% | 46.4% | 33.8% |  |
| Great Egret | 74.6-86.5 | 191-233 | 144-176 | 428.0-490.3 |
| (*n =* 12) | 81.7 ± 3.8 | 213.8 ± 12.6 | 162.2 ± 10.0 | 457.7 ± 22.8 |
|  | 17.8% | 46.7% | 35.4% |  |
| Pacific Reef-Heron | 51.5-63.9 | 99.5-125.3 | 70.2-90.0 | 225.2-279.2 |
| (*n =* 14) | 58.5 ± 3.4 | 111.7 ± 7.5 | 79.9 ± 5.8 | 251.5 ± 15.3 |
|  | 23.3% | 44.4% | 31.8% |  |
| Snowy Egret | 47.4-59.3 | 111.0-144.6 | 75.9-107.1 | 234.3-305.5 |
| (*n =* 14) | 55.0 ± 3.4 | 135.4 ± 10.5 | 96.6 ± 8.2 | 287.0 ± 21.2 |
|  | 19.2% | 47.2% | 33.7% |  |
| Little Blue Heron | 48.9-56.9 | 104.4-137.6 | 76.4-104.1 | 233.5-298.6 |
| (*n =* 12) | 52.7 ± 2.4 | 124.2 ± 9.0 | 91.4 ± 7.1 | 268.2 ± 17.2 |
|  | 19.7% | 46.3% | 34.1% |  |
| Tricolored Heron | 52.0-59.5 | 98.7-143.0 | 86.4-107.4 | 260.0-309.9 |
| (*n =* 14) | 55.9 ± 2.3 | 127.4 ± 10.7 | 97.6 ± 6.1 | 283.2 ± 13.8 |
|  | 19.7% | 45.0% | 34.5% |  |
| Reddish Egret | 64.6-72.8 | 161-188 | 126.9-151.1 | 356.1-410.5 |
| (*n =* 15) | 69.4 ± 2.7 | 175.5 ± 7.2 | 140.4 ± 7.9 | 388.0 ± 16.7 |
|  | 17.9% | 45.2% | 36.2% |  |
| Black Heron | 50.9-55.5 | 113.8-126.7 | 80.6-86.9 | 245.3-267.5 |
| (*n =* 5) | 53.1 ± 1.8 | 121.8 ± 5.1 | 83.7 ± 1.9 | 258.6 ± 8.6 |
|  | 20.5% | 47.1% | 32.4% |  |
| Cattle Egret | 49.8-55.4 | 104.0-123.9 | 72.1-87.1 | 229.8-266.4 |
| (*n =* 12) | 52.6 ± 1.6 | 112.6 ± 5.1 | 76.9 ± 4.7 | 242.2 ± 10.6 |
|  | 21.7% | 46.5% | 31.8% |  |
| Squacco Heron | 47.1-56.3 | 79.6-100.2 | 52.1-65.2 | 178.8-221.7 |
| (*n =* 10) | 52.4 ± 3.8 | 90.3 ± 7.3 | 58.9 ± 4.6 | 201.6 ± 15.5 |
|  | 26.0% | 44.8% | 29.2% |  |
| Green Heron | 46.4-51.4 | 73.6-83.5 | 47.8-52.8 | 169.8-187.0 |
| (*n =* 12) | 49.1 ± 1.7 | 78.9 ± 2.7 | 51.1 ± 1.7 | 179.1 ± 5.7 |
|  | 27.4% | 44.1% | 28.5% |  |
| Agami Heron | 73.1-78.3 | 127.8-140.6 | 99.7-110.7 | 300.6-329.2 |
| (*n =* 3) | 76.2 ± 2.7 | 136.2 ± 7.3 | 106.7 ± 6.1 | 319.1 ± 16.0 |
|  | 23.9% | 42.7% | 33.4% |  |
| Whistling Heron | 53.9-59.3 | 107.9-127.5 | 82.1-95.0 | 246.0-281.8 |
| (*n =* 15) | 56.2 ± 1.6 | 120.7 ± 5.3 | 90.1 ± 3.1 | 267.0 ± 9.1 |
|  | 21.1% | 45.2% | 33.8% |  |
| Capped Heron | 56.7-62.7 | 118.6-138.8 | 87.5-98.8 | 262.8-293.8 |
| (*n =* 12) | 59.5 ± 1.8 | 133.1 ± 5.2 | 93.7 ± 3.5 | 286.3 ± 8.6 |
|  | 20.8% | 46.5% | 32.7% |  |
| Black-crowned Night-Heron | 71.8-82.8 | 123.9-158.7 | 76.5-91.9 | 274.7-320.5 |
| (*n =* 14) | 77.7 ± 2.8 | 132.6 ± 9.0 | 83.7 ± 3.7 | 294.0 ± 13.0 |
|  | 26.4% | 45.1% | 28.5% |  |
| Yellow-crowned Night-Heron | 61.3-78.2 | 116.3-142.9 | 81.4-104.4 | 264.3-311.1 |
| (*n =* 13) | 65.2 ± 4.2 | 131.0 ± 7.3 | 94.8 ± 7.7 | 291.0 ± 14.1 |
|  | 22.4% | 45.0% | 32.6% |  |
| Malayan Night-Heron | 59.4-63.8 | 104.7-110.4 | 70.5-73.5 | 234.6-247.7 |
| (*n =* 2) | 61.6 ± 3.1 | 107.6 ± 4.0 | 72.0 ± 2.1 | 241.2 ± 9.3 |
|  | 25.5% | 44.6% | 29.9% |  |
| Boat-billed Heron | 60.2-70.9 | 107.2-124.2 | 67.8-86.7 | 235.2-281.0 |
| (*n =* 14) | 66.4 ± 3.4 | 115.1 ± 5.4 | 76.5 ± 5.6 | 258.0 ± 13.9 |
|  | 25.8% | 44.6% | 29.6% |  |

^1^Values are range (in mm), mean with standard deviation (in mm), and mean percent (mean ratio x 100) of total leg length for each hind-limb bone per each species. Right column: range (in mm) and mean with standard deviation (in mm) of total leg length per species.
