# Supplementary material for: Morphological Covariance and Onset of Foot Prehensility as Indicators of Integrated Evolutionary Dynamics in the Herons (Ardeidae)
Source: Integr Org Biol. 2023 Mar 22;5(1):obad010. doi: 10.1093/iob/obad010 (PMC10132848; doi:10.1093/iob/obad010)
Supplement: obad010_Supplemental_Files [file obad010_supplemental_files.zip › Heron Morphology.Table S3.IOB-2022-051.R1.docx]

**Table S3** Mass of species of Ardeidae listed in increasing order^1^

| Common name | Scientific name | Mass (g) |
| --- | --- | --- |
| Least Bittern | *Ixobrychus exilis* | 86.3 |
| Zigzag Heron | *Zebrilus undulatus* | 123 |
| Green Heron | *Butorides virescens* | 199.5 |
| Squacco Heron | *Ardeola ralloides* | 287 |
| Black Heron | *Egretta ardesiaca* | 324 |
| Little Blue Heron | *Egretta caerulea* | 339.5 |
| Cattle Egret | *Bubulcus ibis* | 366 |
| Snowy Egret | *Egretta thula* | 371 |
| Tricolored Heron | *Egretta tricolor* | 374.5 |
| Pacific Reef-Heron | *Egretta sacra* | 383 |
| Malayan Night-Heron | *Gorsachius melanolophus* | 414 |
| Whistling Heron | *Syrigma sibilatrix* | 463 |
| Agami Heron | *Agamia agami* | 567 |
| Capped Heron | *Pilherodius pileatus* | 570 |
| Reddish Egret | *Egretta rufescens* | 614 |
| Boat-billed Heron | *Cochlearius cochlearius* | 645 |
| Yellow-crowned Night-Heron | *Nyctanassa violacea* | 682.5 |
| American Bittern | *Botaurus lentiginosus* | 706 |
| Black-crowned Night-Heron | *Nycticorax nycticorax* | 810 |
| Great Egret | *Ardea alba* | 873.5 |
| Bare-throated Tiger-Heron | *Tigrisoma mexicanum* | 1160 |
| Great Blue Heron | *Ardea herodias* | 2295 |
| Cocoi Heron | *Ardea cocoi* | 3200 |
| Goliath Heron | *Ardea goliath* | 4468 |

^1^Data from Dunning (2008)
