# Supplementary material for: Morphological Covariance and Onset of Foot Prehensility as Indicators of Integrated Evolutionary Dynamics in the Herons (Ardeidae)
Source: Integr Org Biol. 2023 Mar 22;5(1):obad010. doi: 10.1093/iob/obad010 (PMC10132848; doi:10.1093/iob/obad010)
Supplement: obad010_Supplemental_Files [file obad010_supplemental_files.zip › Heron Morphology.Fig S1.IOB-2022-051.R1.docx]

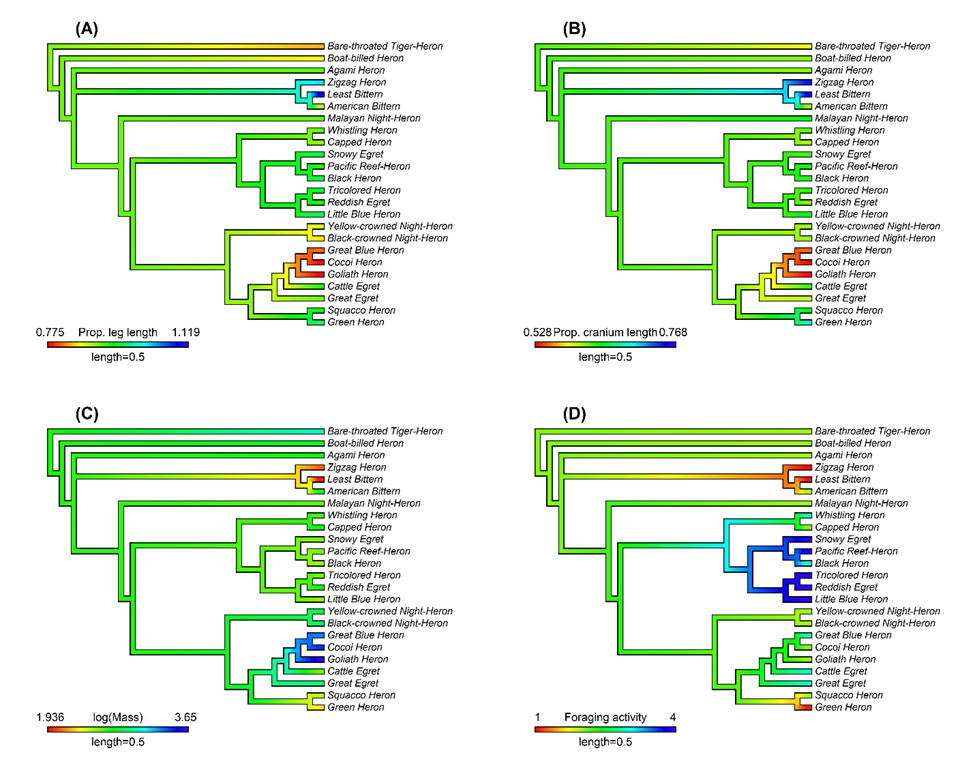


**Fig. S1** Ardeid phylogeny with mapped traits used in this study. For our phylogenetically informed analyses, we based our phylogeny on Hruska (2018). However, a few species in *Ardea* were not included in Hruska’s phylogeny, so we supplemented the information for this genus from Huang et al. (2016). (**A**) Leg length as a proportion of body mass calculated as log (leg length) / log (mass). (**B**) Cranium length as a proportion of body mass calculated as log (cranium length) / log (mass); (**C**) Logarithm of body mass; (**D**) Foraging activity level (1-4). (See text for explanations.)
